# Crocetin and Its Glycoside Crocin, Two Bioactive Constituents From *Crocus sativus* L. (Saffron), Differentially Inhibit Angiogenesis by Inhibiting Endothelial Cytoskeleton Organization and Cell Migration Through VEGFR2/SRC/FAK and VEGFR2/MEK/ERK Signaling Pathways

**DOI:** 10.3389/fphar.2021.675359

**Published:** 2021-04-30

**Authors:** Chen Zhao, Hio-Tong Kam, Yan Chen, Guiyi Gong, Maggie Pui-Man Hoi, Krystyna Skalicka-Woźniak, Alberto Carlos Pires Dias, Simon Ming-Yuen Lee

**Affiliations:** ^1^ State Key Laboratory of Quality Research in Chinese Medicine and Institute of Chinese Medical Sciences, University of Macau, Macao, China; ^2^ Independent Laboratory of Natural Products Chemistry, Department of Pharmacognosy, Medical University of Lublin, Lublin, Poland; ^3^ Centre for the Research and Technology of Agro-Environment and Biological Sciences (CITAB-UM), AgroBioPlant Group, Department of Biology, University of Minho, Braga, Portugal

**Keywords:** crocetin, crocin, angiogenesis, VEGF, zebrafish, HUVEC

## Abstract

Crocetin and crocin are two important carotenoids isolated from saffron (*Crocus sativus* L.), which have been used as natural biomedicines with beneficial effects for improving the suboptimal health status associated with abnormal angiogenesis. However, the anti-angiogenic effects and underlying mechanisms of the effects of crocetin and crocin have not been investigated and compared. The anti-angiogenic effects of crocetin and crocin were tested on human umbilical vein endothelial cells (HUVECs) *in vitro*, and in zebrafish *in vivo*. *In vivo*, crocetin (20 μM) and crocin (50 and 100 μM) significantly inhibited subintestinal vein vessels formation, and a conversion process between them existed in zebrafish, resulting in a difference in their effective concentrations. In the HUVEC model, crocetin (10, 20 and 40 μM) and crocin (100, 200 and 400 μM) inhibited cell migration and tube formation, and inhibited the phosphorylation of VEGFR2 and its downstream pathway molecules. *In silico* analysis further showed that crocetin had a higher ability to bind with VEGFR2 than crocin. These results suggested that crocetin was more effective than crocin in inhibiting angiogenesis through regulation of the VEGF/VEGFR2 signaling pathway. These compounds, especially crocetin, are potential candidate natural biomedicines for the management of diseases associated with abnormal blood vessel growth, such as age-related macular degeneration.

## Introduction

**FIGURE 1:**
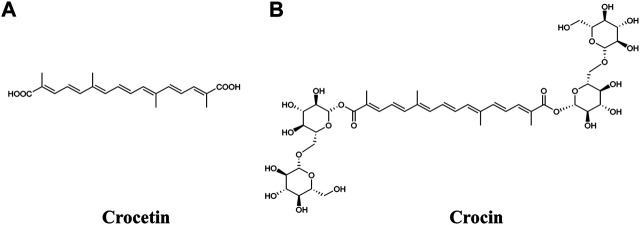
Chemical structures of **(A)** crocetin and **(B)** crocin.

Crocetin and crocin (also known as crocin-I or α-crocin) are two important carotenoids isolated from the dried stigma of the flowers of *Crocus sativus* L. (saffron). Carotenoids have been implicated as playing a versatile role in human health; however, animals (including humans) rarely produce them, and thus need to obtain them *via* the diet (Melendez-Martinez, 2019). One of the most popular applications of saffron in food is as a colorant, with the coloring effect attributed to crocetin and crocin (Bagur et al., 2018; Bian et al., 2020). Crocetin is a lipophilic carotenoid and crocin is the hydrophilic diester of crocetin with gentiobiose (Figure 1). Both compounds have been shown to exhibit a number of biological properties, such as anti-oxidative, anti-inflammatory, anti-lipidemic and anti-tumor activities, and have potential health benefits by modifying different disease processes, including in cardiovascular diseases, metabolic syndromes, ocular disorders and cancer (Alavizadeh and Hosseinzadeh, 2014; Bukhari et al., 2018; Hashemi and Hosseinzadeh, 2019).

In comparison to their anti-hypoxic and anti-tumor effects, only a few previous studies have investigated the effects of crocetin and crocin on angiogenesis. One study reported that crocetin inhibited VEGF-induced tube formation in a co-culture model of human umbilical vein endothelial cells (HUVECs) and fibroblasts, and in a similar fashion inhibited the proliferation and migration of human retinal microvascular endothelial cells (HRMECs) (Umigai et al., 2012). In another study, crocin inhibited HUVEC proliferation and decreased CD34 expression (a marker for endothelial cell differentiation) in tumor tissues in mice (Chen et al., 2019). A previous study also found that crocetin promoted angiogenesis by increasing the cell viability of HUVECs (Nasirzadeh et al., 2019). Although the angiogenic effects of crocetin and crocin in different experimental models have been investigated, their interaction *in vivo*, effective concentrations, and modes of action still need to be studied and compared systematically. In addition, crocetin and crocin have recently been shown to display microtubule-targeting properties that inhibit tubulin assembly and suppress the migration and proliferation of cancer cells (Hire et al., 2017; Sawant et al., 2019; Colapietro et al., 2020). Many microtubule-targeting agents (MTA) are also highly anti-angiogenic due to their abilities to disrupt microtubule dynamics, which play key roles in endothelial cell motility during angiogenic sprouting (Dumontet and Jordan, 2010). However, it is not clear whether crocetin and crocin can inhibit endothelial cell motility or sprouting angiogenesis, nor the difference between them in these respects.

Angiogenesis is a highly regulated process of new blood vessel growth from pre-existing ones. Normal angiogenesis has fundamental roles in physiological conditions, such as wound healing and tissue regeneration, but excessive angiogenesis promotes tumorigenesis and ocular disorders such as age-related macular degeneration (AMD) (Potente et al., 2011; Fallah et al., 2019). AMD is characterized by choroidal neovascularization (CNV), wherein abnormal proliferating blood vessels from the choroidal layer invade the overlaying retina. Vascular endothelial growth factor (VEGF) is secreted in response to oxidative stress and plays important roles in the development of CNV (Ambati and Fowler, 2012). VEGFs are key inducers of angiogenesis that bind with high affinity to receptor tyrosine kinases (VEGFRs), with VEGFA and VEGFR2 being the principal ligand and signaling receptor, respectively, in vascular endothelial cells. The signal transduction network initiated by the VEGFA-VEGFR2 ligand-receptor system leads to the activation of various downstream pathways that play a crucial role in regulating endothelial cell proliferation, survival and migration in the process of angiogenesis (Simons et al., 2016). Anti-VEGF agents are currently used for the treatment of CNV in AMD. Interestingly, a recent study showed that saffron extract ameliorated the retinal degenerative processes in AMD patients, possibly through neuroprotective activities (Di Marco et al., 2019), while another study showed that crocetin prevented retinal pigment epithelia (RPE) from incurring oxidative stress-induced damage and might halt or delay AMD disease progression (Karimi et al., 2020). Additionally, lutein (another carotenoid) has been recommended as a health supplement for AMD patients by the NIH (Melendez-Martinez, 2019). A prospective follow-up study (conducted over two decades) found that the incidence of advanced AMD could be reduced by intake of catenoids (Wu et al., 2015). Compared to lutein, AMD patients could benefit from saffron supplementation to slow down AMD progression (Di Marco et al., 2019). Therefore, it is meaningful to investigate if crocetin and crocin can inhibit VEGF-induced angiogenesis; this will provide insight facilitating the discovery and development of new agents for the treatment of AMD.

In the present study, we determined the effective dose ranges of crocetin and crocin for inhibiting sprouting angiogenesis in *Tg(fli1:EGFP)* zebrafish. Since orally administered crocin was previously reported to be transformed into crocetin in rat (Zhang et al., 2017), we also examined the metabolism of crocetin and crocin in zebrafish by using high-performance liquid chromatography (HPLC). Furthermore, we investigated the underlying molecular mechanisms of the anti-angiogenic effects of crocetin and crocin in HUVECs *in vitro*.

## Materials and Methods

### Ethics Statement

All animal experiments were conducted according to the ethical guidelines of ICMS, University of Macau and the protocol was approved by ICMS, University of Macau (UMARE-303-2017)

### Chemicals, Regents, Cell Lines and Animals

Crocetin and crocin (purity by HPLC ≥98.0%) were purchased from Sichuan Weikeqi Biotech Co., Ltd (Sichuan, P.R. China). Crocetin and crocin were dissolved in dimethylsulfoxide (DMSO) as 10 mM and 100 mM stock solution, respectively. All of the stock solutions were stored at −20°C and diluted into different concentrations in appropriate assay media as required.

Kaighn’s modification of Han’s F12 medium (F-12 K), fetal bovine serum (FBS), penicillin-streptomycin (P/S), phosphate-buffered saline (PBS) and 0.25% (w/v) trypsin/1 mM EDTA were purchased from Invitrogen (Carlsbad, CA, United States). Endothelial cell growth supplement (ECGS), heparin, gelatin and dimethyl sulfoxide (DMSO) were purchased from Sigma-Aldrich Co. (St. Louis, MO, United States). Cell Proliferation Kit II (XTT) was obtained from Roche, Mannheim, Germany. VEGF was obtained from R&D Systems (Minneapolis, MN, United States). Growth factor reduced (GFR) Matrigel™ was supplied by BD Biosciences (Bedford, MA, United States). VEGF receptor (VEGFR) tyrosine kinase inhibitor II (VRI) was obtained from CalbioChem (Merck, Germany) and SU5416 was obtained from Sigma Aldrich Co. The following antibodies were used: ERK1/2 (Cell Signaling Technology, Danvers, MA, United States; Cat# 9102S), phospho-ERK1/2 (Thr202/Tyr204) (Cell Signaling Technology; Cat# 9101S), SRC (Cell Signaling Technology; Cat# 2109S), phospho-SRC (Tyr527) (Cell Signaling Technology; Cat# 2105S), MEK (Cell Signaling Technology; Cat# 9122L), phospho-MEK (Ser217/221) (Cell Signaling Technology; Cat# 9121S), FAK (Cell Signaling Technology; Cat# 3285S), phospho-FAK (Tyr576/577) (Cell Signaling Technology; Cat# 3281S), VEGFR2 (Cell Signaling Technology; Cat# 2472S), phospho-VEGFR2 (Tyr1175) (Cell Signaling Technology; Cat# 2478L) and GAPDH (Cell Signaling Technology; Cat# 2118L). The HPLC-grade methanol, formic acid and acetonitrile used in the HPLC analysis were provided by Merck (Darmstadt, Germany).

HUVECs were obtained from Invitrogen. Transgenic zebrafish *Tg(fli1:EGFP)* expressing enhanced green fluorescent protein (EGFP) under the control of fli1 promoter were provided by the Zebrafish Information Network (ZFIN, Eugene, OR, United States).

### Maintenance of Zebrafish and Their Embryos

Transgenic zebrafish *Tg(fli1:EGFP)* and wild-type zebrafish were maintained as described in the Zebrafish Handbook. Adult zebrafish were kept in a controlled environment at 28.5°C, under a 14 h light/10 h dark cycle. They were fed general tropical fish food once daily and live brine shrimp twice daily. Zebrafish embryos were generated by natural pairwise mating and collected to be raised in embryo media at 28.5°C in an incubator. Dead and unfertilized embryos were picked out at 4 hours post fertilization (hpf), and embryos were distributed into a multi-plate with 8–12 embryos in each group depending on the assay at 24 hpf.

### Zebrafish Embryo Morphological Observations

Normally developing *Tg(fli1:EGFP)* zebrafish embryos were digested with 1 mg/ml of protease at 24 hpf, and then distributed into a 24-well plate with 10 embryos per group. Each group was incubated with 1 ml embryo water containing different concentrations of compounds for an additional 48 h. Embryos receiving DMSO (0.1%) served as vehicle control, and those receiving 50 ng/ml of VRI served as a positive control. The subintestinal vessels (SIVs) of embryos were observed and imaged at 72 hpf under an Olympus Spinning Disk Confocal Microscope System (IX81 Motorized Inverted Microscope [w/ZDC], IX2 universal control box, X-cite series 120, DP71 CCD Camera; Olympus, Tokyo, Japan). Images were captured at 40× and 100× magnifications. The total area (A) of SIVs was quantified with ImageJ (NIH, Bethesda, MD, United States) and the SIV inhibition rate was calculated using the following formula:

$$SIV\;Inhibition\%\;=1-\frac{A\left(Drug\;treatment\right)}{A\left(Vehicle\right)}\times 100\%$$

### Metabolic Analysis of Zebrafish Larvae by HPLC

*Tg(fli1:EGFP)* zebrafish embryos were digested with 1 mg/ml of protease, and then distributed into a 12-well plate with 30 embryos per group in 24 hpf. Zebrafish larvae were treated with incubation medium (vehicle control), crocetin (20 μM) and crocin (100 μM) for 48 h and then collected to determine the metabolism of crocin and crocetin. After washing three times with Milli-Q water, zebrafish larvae were homogenized with 100 μL methanol in a 1.5 ml centrifuge tube. All samples were centrifuged at 15,000 g for 15 min, and the supernatants were obtained and subsequently subjected to HPLC analysis with a XBridge™ C-18 column (5 μm, 4.6 × 250 mm). The column temperature was set at 30°C and the injection volume was 10 μL. By comparison with and optimization based on published studies, the detection wavelength was selected at 440 nm (Chryssanthi et al., 2011; Mary et al., 2016). The mobile phase consisted of 0.1% formic acid in water (A) and acetonitrile (B); the flow rate was set at 1 ml/min. A gradient elution program was set as follows: 0–9 min, 20-50% B; 9–11 min, 50–70% B; 11–20 min, 70–95% B; 20–24 min, 95% B; 24–30 min, 95-20% B at a flow rate of 1.0 ml/min.

### Cell Culture

HUVECs were cultured in F-12K medium supplemented with 100 g/ml heparin, 30 g/ml ECGS, 10% FBS and 1% P/S at 37°C in a humidified atmosphere with 5% CO_2_ (v/v). Early passages (3–7 passages) were used in all assays.

### Cell Viability Assay

HUVECs (1×10^4^ cells/ml) were seeded into 96-well plate in F-12K complete medium for 24 h for attachment. Then, the cells were treated with various concentrations of crocetin and crocin in low serum media (0.5% FBS) for 24 h. Cells receiving 0.1% DMSO served as vehicle control. Cell viability was assessed by using XTT assay, as described previously (Lam et al., 2012). Absorbance was measured using a Microplate Reader (Molecular Devices, San Jose, CA, United States) at wavelengths of 490 and 650 nm. For each compound, three independent experiments were conducted.

### *In Vitro* Wound Healing Assay

HUVECs in growth medium were seeded into 24-well plates and grown to confluence. A wound area was created on the monolayer cells by scratching with 200 μL pipette tips. Non-adherent cells were removed by washing with PBS, and high serum (10% FBS) medium containing various concentrations of compounds, 0.1% DMSO (vehicle control) and SU5416 of 10 μM (positive control) was added to each well. After 20 h incubation, cells were washed with PBS. Images were taken at 0 and 20 h independently using an inverted light microscope (IX73 Motorized Inverted Microscope; Olympus). Images were analyzed by ImageJ, which is able to analyze the ability for cell migration by calculating the wound area. The distance migrated was calculated and analyzed by Image Pro-Plus 6.0. The values were observed from three randomly selected fields. The relative inhibition rate was expressed relative to the vehicle control group.

### *In Vitro* Capillary-Like Tube Formation Assay

Capillary-like tube formation assay was performed using HUVECs as described previously (Li et al., 2020). GFR Matrigel was thawed at 4°C overnight. A pre-chilled 96-well plate was coated with 50 μL Matrigel (for each well) and incubated at 37°C for 30 min for solidification. HUVECs resuspended and diluted at a density of 1×10^4^ per well, in low serum (0.5% FBS) F-12K medium containing the indicated concentrations of agents, were seeded onto the Matrigel-coated 96-well plate and incubated at 37°C. After 6 h, capillary-like tubes were formed in the vehicle control group; then, cells were stained with 1 mM Calcein AM (Life Technologies, Carlsbad, CA, United States) for 30 min at 37°C. Images were captured at 4× magnification under a fluorescent inverted microscope (IX73 Motorized Inverted Microscope; Olympus). Capillary-like tube formation was quantified by measuring tube length in three randomly selected fields by ImageJ.

### Western Blot Assay

HUVECs were seeded in 6-well plates and incubated overnight for confluence. After starving in low serum (0.5% FBS) F-12K medium for 2 h, HUVECs were treated with various concentrations of compounds, 0.1% DMSO and SU5416 (10 μM) for 4 h before stimulating with 50 ng/ml VEGF for 15 min. Then, cells were rinsed with PBS and lyzed in RIPA buffer with the addition of cocktail and PMSF. The concentration of protein extracts was quantified with a BCA Protein Kit according to the protocol described by the manufacturer. Protein (30 μg) was denatured for 5 min at 95°C and subjected to 10% SDS-PAGE. After electrically transferring the proteins to PVDF membranes, they were blocked with 5% non-fat milk in TBS-0.1% Tween 20 (TBST) for 1 h at room temperature, and then incubated with primary antibodies of ERK1/2, phospho-ERK1/2, FAK, phospho-FAK, MEK, phospho-MEK, SRC, phospho-SRC, VEGFR2, phospho-VEGFR2, and GAPDH at 4°C overnight. After washing with TBST, membranes were incubated with horseradish peroxidase-conjugated goat anti-rabbit antibody (Beyotime, Shanghai, China) for 1 h at room temperature. After repeated washing with TBST, immunoreactive bands of proteins were visualized using an ECL advanced Western blotting detection kit. Images of the protein bands were taken using Image Lab (Bio-Rad, Hercules, CA, United States). The density of each band was measured by Bio-Rad Image 3.0, and the ratios of phosphorylated protein/total protein were calculated in corresponding bands from the same blot.

### Molecular Docking Analysis

Molecular docking was employed to explore molecular interactions between VEGFR2 and crocetin or crocin. The X-ray crystallography structure of VEGFR2 (PDB ID: 5EW3) (Bold et al., 2016) was retrieved from the RCSB Protein Data Bank (http://www.rcsb.org/pdb). Co-crystallized ligand and crystal water molecules were removed from the protein structure, and the nonpolar hydrogen atoms were added. A gridbox was created to enclose VEGFR2, allowing us to find the most suitable binding site of crocetin or crocin. The best docking results were selected based on their estimated protein−ligand complex binding free energy and are presented in the present study. PyMOL 1.8 was used to analyze and visualize the molecular interactions between each compound and VEGFR2 (Salentin et al., 2015).

### Data and Statistical Analysis

All values are presented as mean ± SD of three independent experiments. Data were analyzed by GraphPad Prism 7.0 (GraphPad Software Inc., La Jolla, CA, United States). The statistical significance of data was evaluated using one-way ANOVA followed by Dunnett’s multiple comparison test. *p* values less than 0.05 were considered statistically significant.

## Results

### Crocetin and Crocin Exhibited Anti-angiogenic Effects in *Tg(fli1:EGFP)* Zebrafish

**FIGURE 2:**
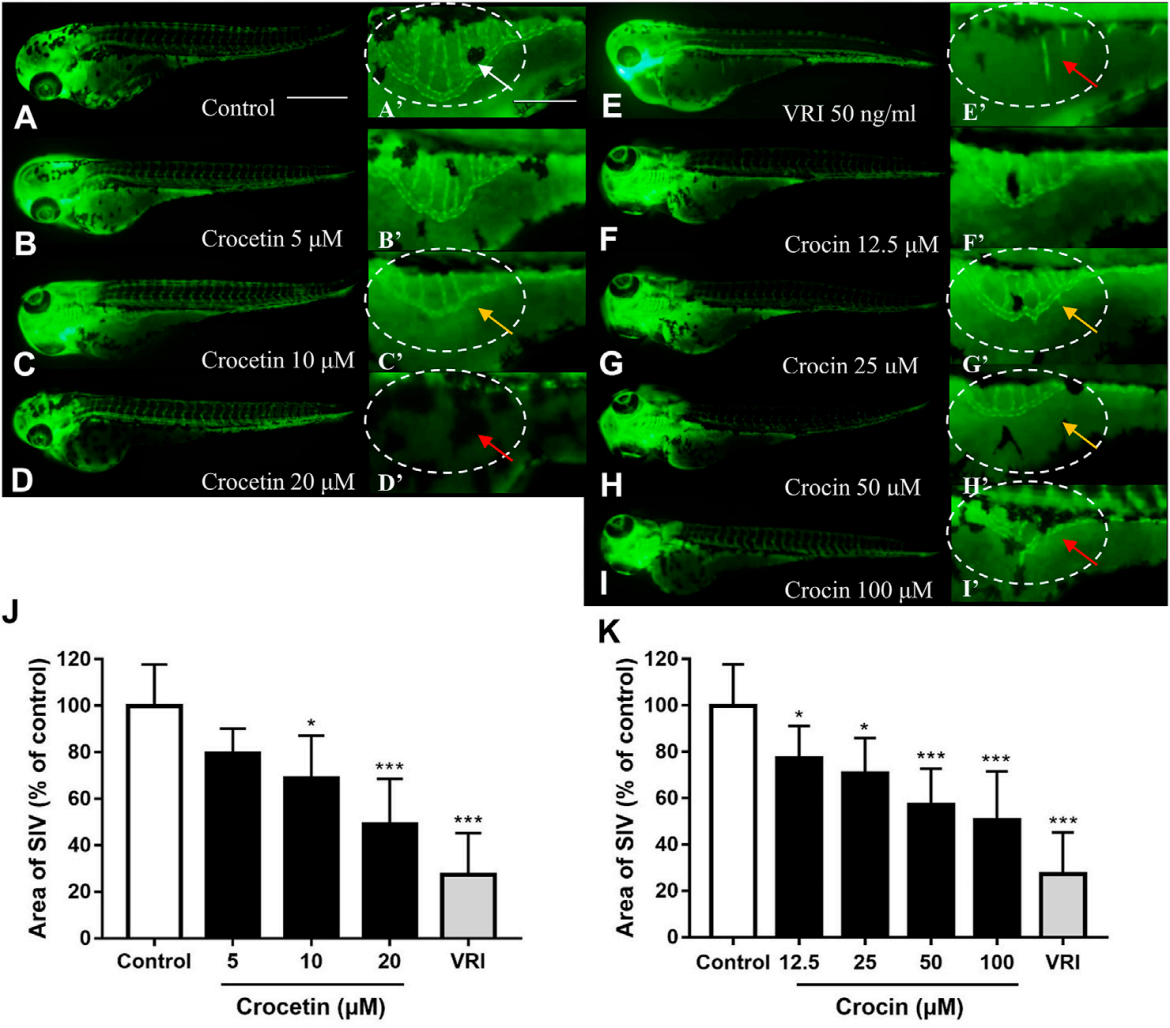
Crocetin and crocin inhibited SIV formation in *Tg(fli1:EGFP)* zebrafish embryos. Zebrafish embryos at 24 hpf were treated by **(A,A’)** 0.1% DMSO (Control) **(E,E’)** 50 ng/ml VRI, **(B-D,B’-D’)** crocetin, or **(F–I,F’–I’)** crocin for 48 h. **(A’–I’)** Magnified views (100× magnification) of the panels labelled A to I (40× magnification). Mature SIVs are indicated by white arrows. Moderately defective SIVs are indicated with yellow arrows and severely defective SIVs are indicated with red arrows (J and K) Quantification of the total area of SIVs reduced by crocetin and crocin. Data are percentages of the control, measured as means ± SD (10 zebrafish embryos per well from three time-independent experiments, *n* = 3). Scale bar = 200 μm. Statistical analysis was performed by one-way ANOVA followed by the Dunnett’s test. *p<0.05 and ***p<0.001 versus control group.

Transgenic zebrafish *Tg(fli1:EGFP)* embryos showing green fluorescent protein expression in vascular endothelial cells under the control of promoter fli1 (Lawson and Weinstein, 2002) were used to investigate the effects of crocetin and crocin on angiogenesis *in vivo*. The process of SIV formation angiogenesis in zebrafish embryo is widely used as a visual guide when inspecting and evaluating of pro- and anti-angiogenic agents. Zebrafish embryos (24 hpf) were incubated with crocetin or crocin for 48 h, and the structure of SIVs was examined. As shown in Figure 2A’, at 72 hpf the vascular plexus of SIVs formed a smooth basket-like structure arranged in an orderly manner in the control group. The crocetin and crocin treatments significantly reduced the vasculature formation of SIVs (indicated by yellow arrows in Figures 2C’,G’,H’). At higher concentrations of crocetin (20 μM) and crocin (100 μM), the structure of the vascular plexus became defective or nearly absent (indicated by red arrows in Figures 2D’,I’). Quantification of the total area of SIVs showed that both crocetin and crocin significantly reduced the formation of SIVs in a concentration-dependent manner, and the maximal effects were comparable to VRI, a strong VEGFR inhibitor that greatly inhibits SIVs angiogenesis (Li et al., 2014).

### Metabolism of Crocetin and Crocin in Zebrafish Larvae

**FIGURE 3:**
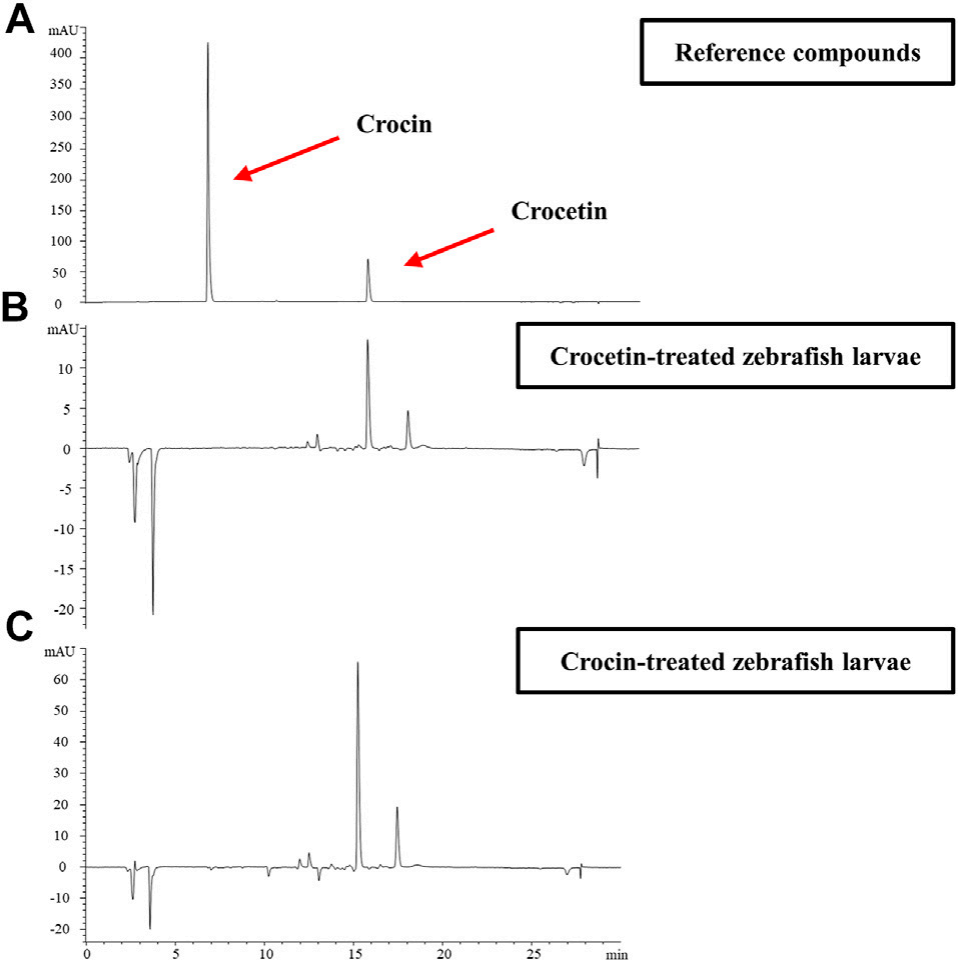
Representative HPLC profiles for **(A)** reference compounds of crocetin and crocin **(B)** Compounds detected in zebrafish larvae homogenates collected within 72 hpf after crocin treatment (100 μM) **(C)** Compounds detected in zebrafish larvae homogenates collected within 72 hpf after crocetin treatment (20 μM).

We further investigated the metabolism of crocetin and crocin in zebrafish larvae by using HPLC. Figure 3A shows that the retention times of crocetin and crocin were 6.570 and 15.223°min, respectively with regard to the reference compound profiles. Interestingly, after the administration of crocin to zebrafish larvae, the peak for crocin could not be detected, although the peak for crocetin was detected (Figure 3B). In the case of crocetin administration, only crocetin was detected (Figure 3C). This result suggested that a conversion process between crocetin and crocin existed in the zebrafish larvae. Given that crocetin is recognized as the bio-active compound produced by converting crocin (Xi et al., 2007; Zhang et al., 2017), we speculated that crocetin was the active compound responsible for the anti-angiogenic effects in zebrafish.

### Effects of Crocetin and Crocin on Endothelial Cell Viability

**FIGURE 4:**
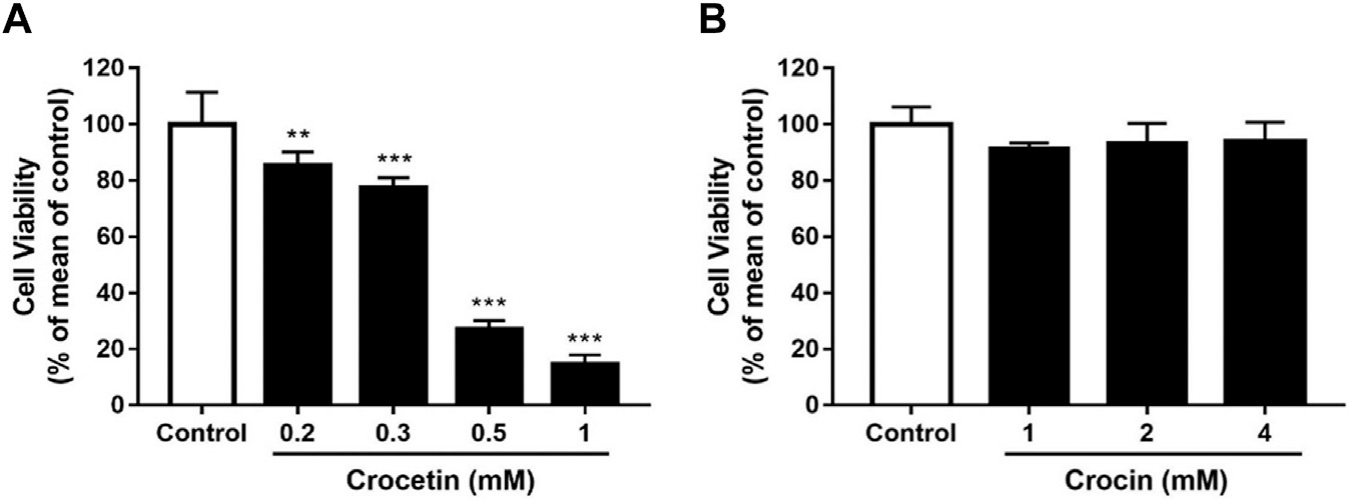
Cytotoxicity of crocetin and crocin on HUVECs (A) HUVECs were treated with different concentrations of crocetin (0.2, 0.3, 0.5, and 1 mM) and **(B)** crocin (1, 2 and 4 mM) for 24 h and examined by XTT assay. The IC_50_ value of crocetin **(A)** is 372.6 μM. Data are means ± SD of three independent experiments (*n* = 3) as a percentage of control. Statistical analysis was performed by one-way ANOVA followed by Dunnett’s test. **p<0.01; ***p<0.001 versus control group.

To evaluate the effects of crocetin and crocin on endothelial cell viability, HUVECs were treated with increasing concentrations of crocetin (0.2, 0.3, 0.5, and 1 mM) and crocin (1, 2, and 4 mM) for 24 h followed by assessment with XTT assay. Crocetin inhibited cell viability in a concentration-dependent manner with an IC_50_ of 372.6 μM (Figure 4A), whereas crocin showed no obvious inhibitory effect up to 4 mM (Figure 4B).

### Crocetin and Crocin Inhibited Migration and Tube Formation in HUVECs

**FIGURE 5:**
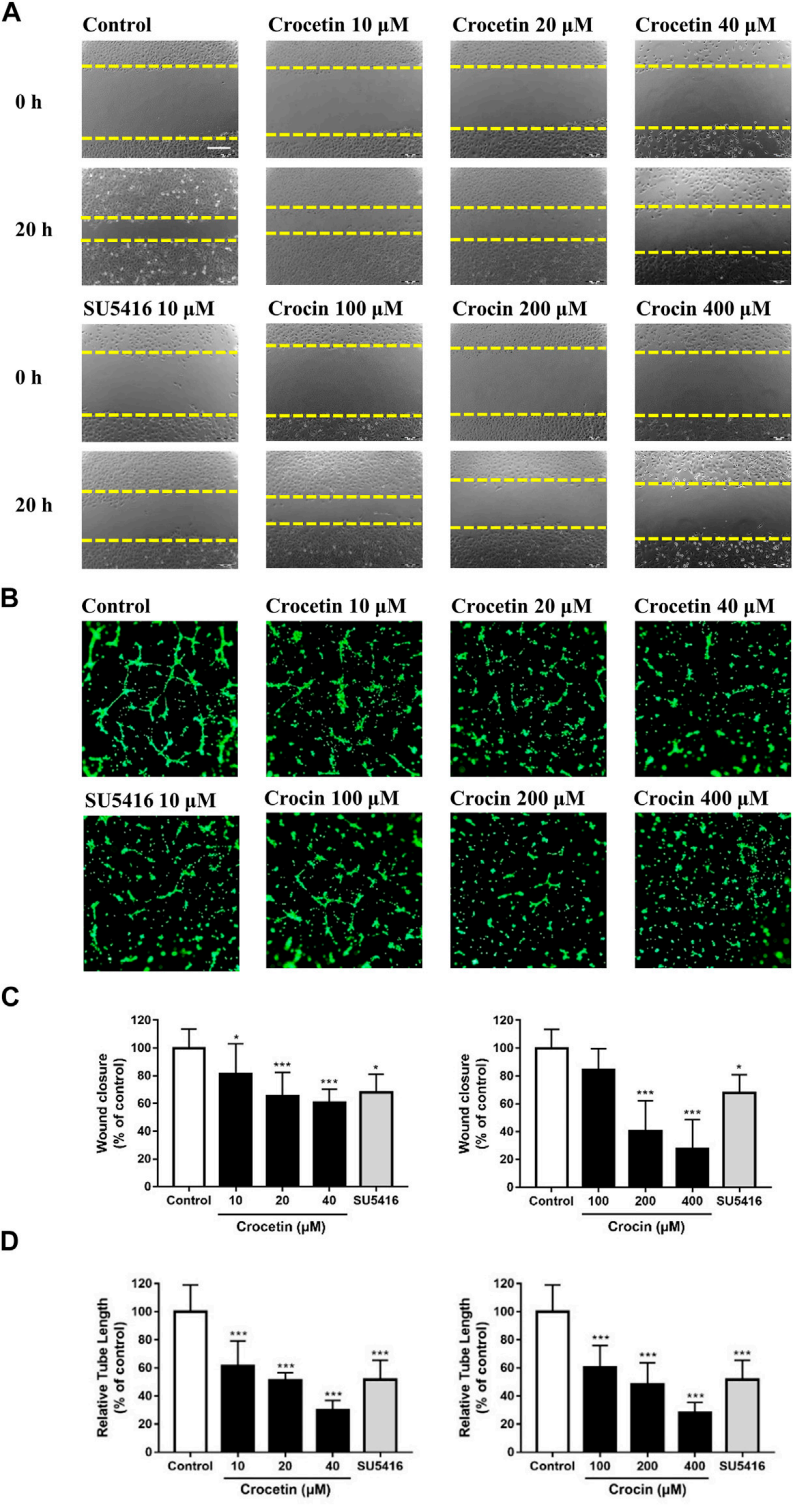
Crocetin and crocin inhibited endothelial cell migration and capillary-like tube formation. **(A)** Wound healing assay for HUVEC migration after 20 h of incubation with vehicle control or drug treatments. **(B)** The migratory ability was evaluated by measuring the mean length of the scraped area of each well and comparing it to the control group. Yellow dashed lines indicated the wound edges. **(C)** Morphological features of the capillary-like tube formation of HUVECs in Matrigel after 6 h of incubation with vehicle control or drug treatments. **(D)** The tube formation ability was evaluated by measuring the total tube length of HUVECs and comparing it to the control group. Scale bar = 200 μm. Data are means ± SD of three independent experiments. Statistical analysis was performed by one-way ANOVA followed by Dunnett's test. *p<0.05 and ***p<0.001 versus control group.

Angiogenesis is a complex process that involves endothelial cell migration and alignment to form tubular structures. We determined the effects of crocetin and crocin on endothelial cell migration and capillary-like formation using the wound healing assay and tube formation Matrigel model. Figures 5A,B showed that there was significant migration of HUVECs to the scraped area in the vehicle control group 20 h after wounding. Crocetin caused 18.8, 34.8, and 39.2% reductions in HUVEC migration at 10, 20, and 40 μM, respectively. Similarly, crocin caused 15.5%, 59.7%, and 72.3% reductions in HUVEC migration at 100, 200, and 400 μM, respectively. Figures 5C,D showed that when HUVECs were cultured on Matrigel in the vehicle control, they aligned and formed capillary-like tube structures after 6 h, and both crocetin and crocin inhibited the morphogenetic changes in tube formation in HUVECs. Crocetin caused 38.6, 48.8, and 70.1% reductions in tube length at 10, 20, and 40 μM, respectively. Similarly, crocin caused 39.3, 51.6, and 71.8% reductions in tube length at 100, 200, and 400 μM, respectively. Statistical analysis of the quantitative measurements showed that both crocetin and crocin induced significant reductions in HUVECs migration and tube formation in concentration-dependent manners. The effects of crocetin and crocin were comparable to SU5416 (a selective inhibitor of VEGFR2) (Litz et al., 2004).

### Crocetin and Crocin Inhibited the Activation of Key Proteins Involved in Angiogenesis Signaling in HUVECs

**FIGURE 6:**
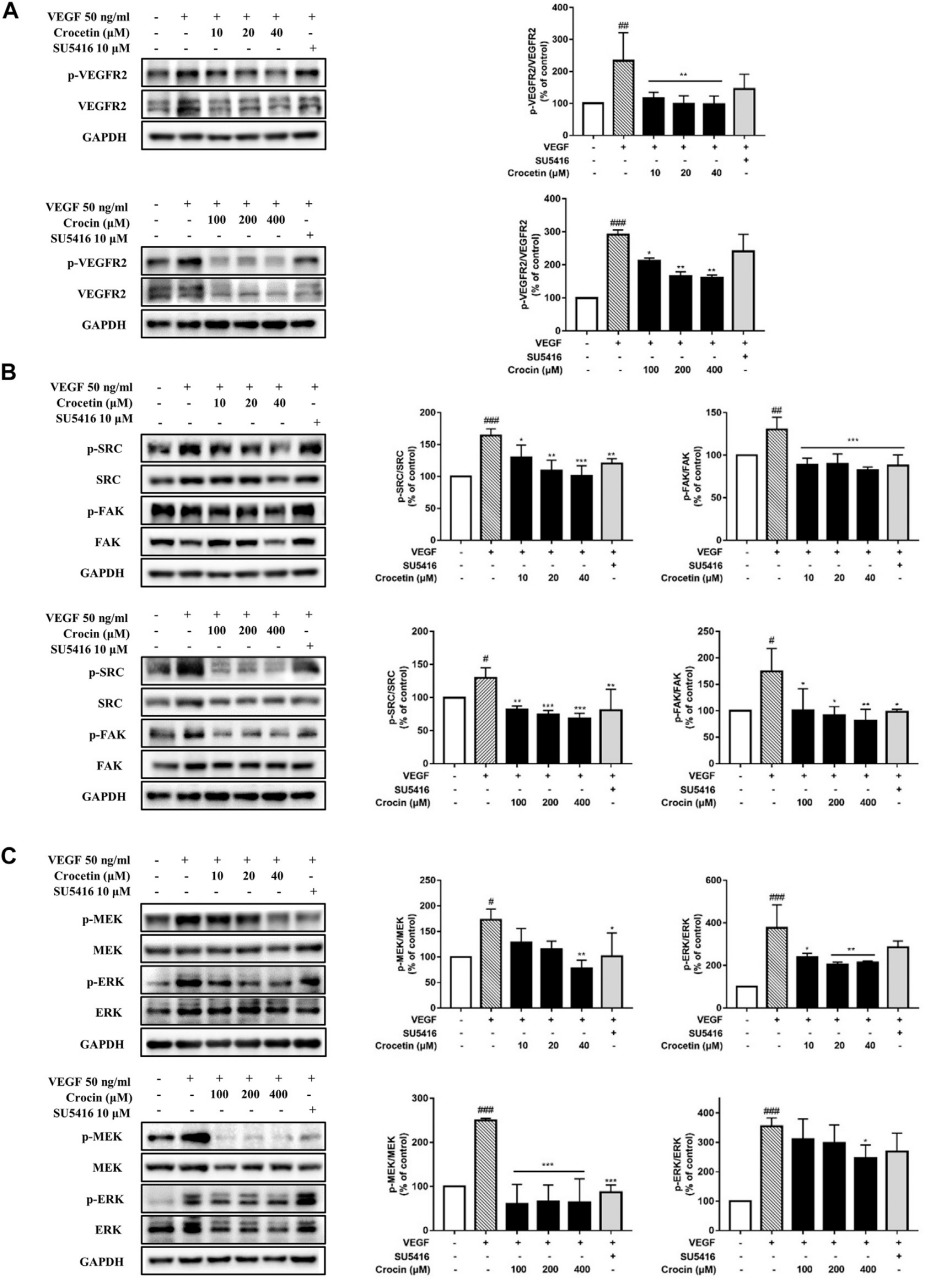
Crocetin and crocin inhibited the activation of VEGFR2 and its downstream signaling pathways. HUVECs were starved for 2 h and then pretreated with crocetin (10, 20 and 40 μM) or crocin (100, 200 and 400 μM) for 4 h before being stimulated by VEGF (50 ng/ml) for 15 min. Western blot assay was used for investigating the expression levels of the major proteins involved in VEGF-mediated angiogenesis signaling in HUVECs. Crocetin and crocin down-regulated the expression levels of **(A)** p-VEGFR2 **(B)** p-SRC and p-FAK, and **(C)** p-MEK and p-ERK. Protein expression levels were quantified by densitometry. Results are percentages relative to control; means ± SD of three independent experiments (*n* = 3) are shown. Statistical analysis was performed by one-way ANOVA followed by Dunnett’s test. *p<0.05; **p<0.01 and ***p<0.001 versus VEGF treatment group. #p<0.05; ##p<0.01 and ###p<0.001 versus control group.

To investigate the possible mechanisms underlying the anti-angiogenic effects of crocetin and crocin in HUVECs, protein expression levels were determined for several key proteins involved in the regulation of angiogenesis by Western blot. As shown in Figure 6, crocetin (10, 20, and 40 μM) and crocin (100, 200 and 400 μM) concentration-dependently inhibited the upregulation in protein expression levels of p-VEGFR2 induced by VEGF (50 ng/ml), as well as the downstream signaling kinases p-SRC, p-FAK, p-MEK, and p-ERK. Notably, the two pathways (VEGFR2/MEK/ERK and VEGFR2/MEK/ERK) showed different sensitivity to the inhibitory effects of crocetin and crocin. At 10 μM, crocetin had no effect on MEK phosphorylation; however, reduced the protein levels of p-SRC, p-FAK, and p-ERK. And crocetin reduced the protein levels of p-SRC and p-FAK more effectively than those of p-ERK at 40 μM. In the case of crocin, high concentrations significantly reduced p-SRC, p-FAK, and p-MEK, and to a lesser extent p-ERK. The VEGFR2/SRC/FAK pathway is mainly involved in focal adhesion turnover, cell shape and migration, and the VEGFR2/MEK/ERK pathway is mainly involved in endothelial gene transcription and proliferation (Simons et al., 2016; Fallah et al., 2019). Thus, these results suggested that crocetin and crocin might effectively inhibit angiogenesis by inhibiting endothelial cytoskeleton organization and cell migration *via* regulation of VEGFR2/SRC/FAK, and to a lesser extent, VEGFR2/MEK/ERK signaling.

### Molecular Docking Studies of Crocetin and Crocin With VEGFR2

**FIGURE 7:**
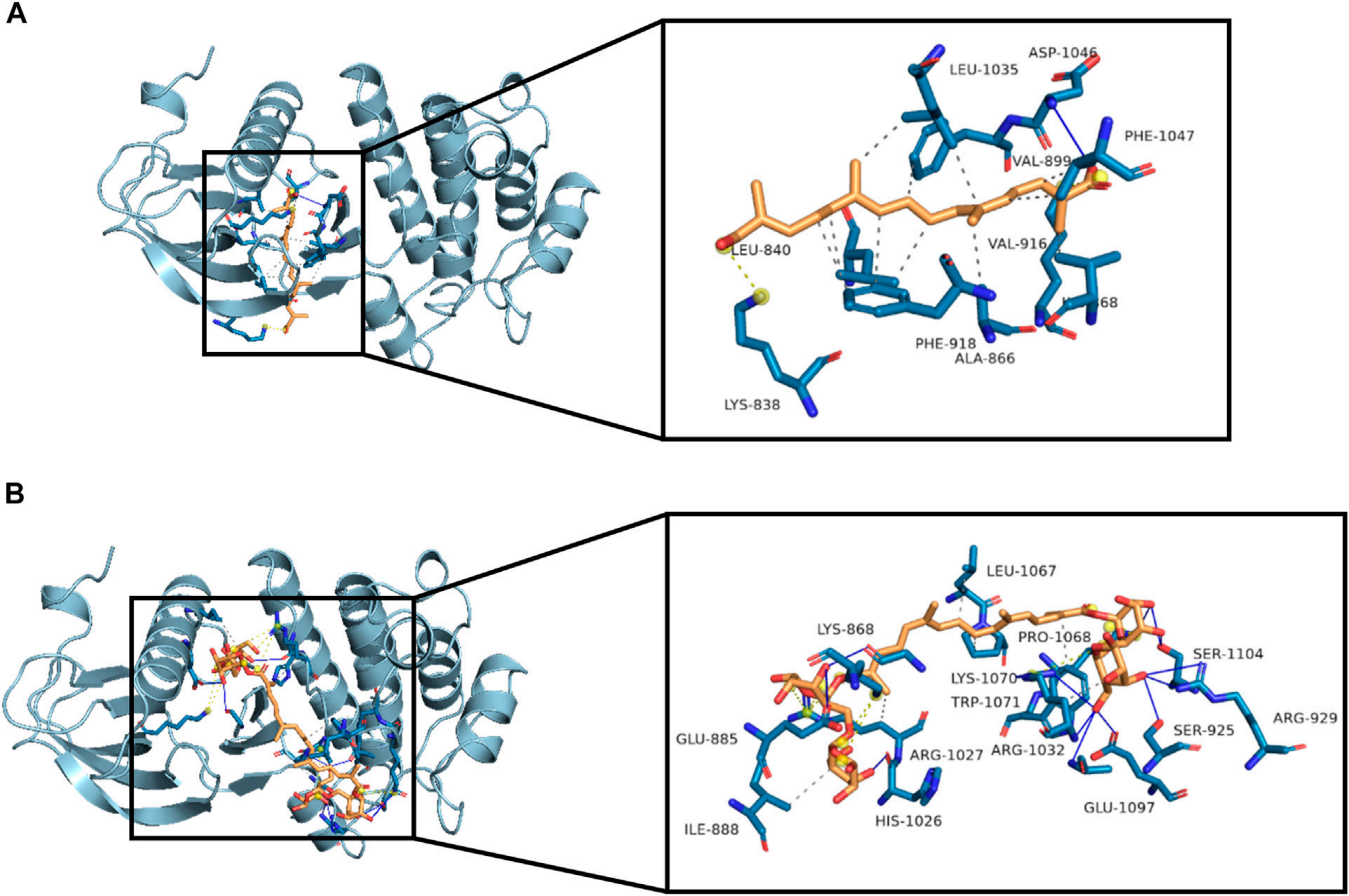
Molecular interactions between crocetin or crocin with VEGFR2. Three-dimensional view of crocetin and crocin located in the binding site of VEGFR2 are shown in **(A)** and **(B)**, with affinities of −8.6 and −8.4 kcal/mol, respectively. Hydrophobic interactions (dashed lines), hydrogen bonds (blue lines) and important residues of the binding site are shown.

Since crocetin and crocin down-regulated p-VEGFR2 and its downstream signaling kinases differently, molecular docking studies were carried out to investigate the binding sites between crocetin or crocin and VEGFR2. The binding site of VEGFR2 was defined by a co-crystallized compound (AAL993) (Bold et al., 2016). Crocetin was well docked with the binding site of VEGFR2 (Figure 7A) through hydrophobic interactions and a hydrogen bond, with an affinity of −8.6 kcal/mol. In addition, crocetin was trapped in a hydrophobic pocket, which was composed of Leu 840, Ala 866, Val 899, Val 916, Phe 918, Leu 1035 and Phe 1047. A hydrogen bond was also generated between crocetin and the key residue Asp 1046. In comparison, crocin bound to a different site than the co-crystallized compound (AAL993) *via* hydrophobic interactions and hydrogen bonds, with an affinity of −8.4 kcal/mol (Figure 7B). Although the computed Gibbs free energy values of crocetin and crocin were similar, their predicted binding sites to VEGFR2 were different. It appears that crocin did not have a stable interaction with VEGFR2. These results suggested that crocetin showed more ability to bind with VEGFR2 than crocin, which might explain their different anti-angiogenic effects.

## Discussion

Saffron is widely used as a natural spice against many diseases (Shahi et al., 2016). Since crocetin and crocin are two important carotenoids derived from saffron and have been used for centuries, it is useful to investigate and compare the difference in their therapeutic values and biological effects. The present study represented the first detailed investigation of the anti-angiogenic activities of crocetin and crocin, and used transgenic *Tg(fli1:EGFP)* zebrafish embryos *in vivo* and endothelial cell model HUVECs *in vitro*.

We evaluated the effects of crocetin and crocin by using a *Tg(fli1:EGFP)* zebrafish model *in vivo*, which allowed for direct observation of angiogenesis in real-time (Lawson and Weinstein, 2002; Gong et al., 2016). Our group has previously identified several natural compounds that exhibited anti-angiogenic effects by using the zebrafish model, such as indirubin (Alex et al., 2010), citrus flavonoids (Lam et al., 2012) and an andrographolide derivative (Li et al., 2020). In the present study, we observed that there was no obvious toxicity in zebrafish embryos treated with crocetin and crocin (Supplementary Figure S1). More importantly, crocetin and crocin inhibited SIV formation in a concentration-dependent manner (Figure 2). Moreover, the effective concentrations of crocetin (5, 10 and 20 μM) were lower than those of crocin (12.5, 25, 50 and 100 μM), which led us to investigate whether the different anti-angiogenic responses between crocetin and crocin might be caused by their metabolic characteristics. Therefore, we evaluated the metabolism of crocetin and crocin in zebrafish larvae after the drug administration using HPLC. It was interesting to note that there was a conversion between crocetin and crocin in zebrafish *in vivo* (Figure 3). This finding was in agreement with a previous study indicating that crocetin might be the active metabolite of crocin in rats (Zhang et al., 2017). Therefore, we postulated that crocetin was the active metabolite of crocin responsible for inducing the anti-angiogenic effect in zebrafish.

**FIGURE 8:**
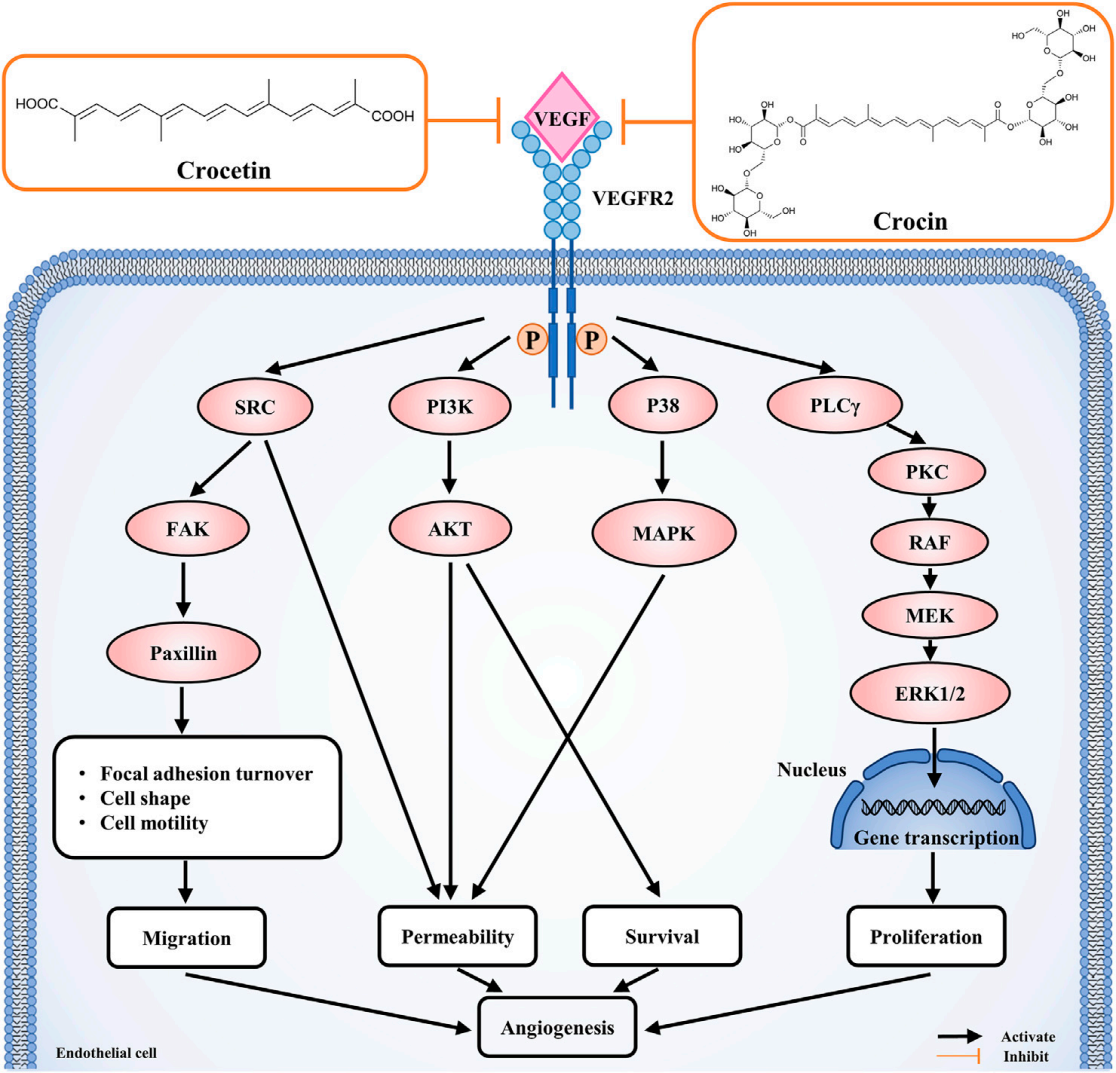
Crocetin and crocin inhibited angiogenesis *via* the inhibition of VEGFR2 signaling and downstream SRC, FAK, MEK, ERK kinase activation.

Given the differences in metabolism and anti-angiogenic activities of crocetin and crocin in zebrafish, their mechanisms for inhibiting angiogenesis were further investigated in HUVECs *in vitro*. Several recent studies suggested that saffron extract and/or crocetin might halt or delay disease progress in AMD. It has been shown that saffron extract was effective at ameliorating the retinal degenerative processes in AMD patients, possibly through neuroprotective activities (Di Marco et al., 2019), while crocetin prevented RPE from incurring oxidative stress-induced damage (Karimi et al., 2020). In addition, clinical studies showed that saffron supplementation could improve retinal function in AMD patients (Falsini et al., 2010; Piccardi et al., 2012). AMD is characterized by CNV, wherein VEGF is secreted in response to oxidative stress and induces abnormal angiogenesis from the choroidal layer to the overlaying retina (Ambati and Fowler, 2012). Currently, anti-VEGF therapies with bevacizumab (monoclonal anti-VEGF antibody) and pegaptanib (anti-VEGF aptamer) are FDA-approved for treating AMD (Solomon et al., 2014). Our result showed that crocetin (10, 20 and 40 μM) and crocin (100, 200 and 400 μM) were effective anti-angiogenic agents that significantly inhibited HUVEC migration in wound healing assay, as well as tube formation in a Matrigel model in a concentration-dependent manner (Figure 5). The cytotoxicity of crocetin (0.2 mM) was higher than that of crocin (>4 mM) in HUVECs (Figure 4). In line with our results, a previous study (Umigai et al., 2012) reported that crocetin inhibited proliferation and tube formation in a HUVEC and fibroblast co-culture. They also showed that crocetin inhibited VEGF-induced proliferation and migration of HRMECs *via* the inhibition of p38 activation (Umigai et al., 2012). Here, we demonstrated that crocetin and crocin inhibited VEGFR2 signaling and suppressed downstream p-SRC, p-FAK, p-MEK, and p-ERK activation in HUVECs (Figure 8), but we did not observe suppression of p38 (data not shown). In endothelial VEGF signaling networks, SRC and FAK are tyrosine kinases that play crucial roles in cytoskeletal reorganization, cell motility and migration, while MEK/ERK signaling is a well-studied pathway that mainly regulates endothelial proliferation among other important processes in angiogenesis, including survival, differentiation and migration (Simons et al., 2016; Fallah et al., 2019). Moreover, crosstalk between MEK/ERK and SRC/FAK is also observed in the regulation of VEGF-mediated angiogenesis (Hood et al., 2003). Activation of the ERK pathway by crocetin seems to be important for improving AMD; in a recent study (Karimi et al., 2020), crocetin prevented tert-butyl hydroperoxide (TBHP)-induced oxidative stress in RPE cells by activating the ERK pathway to preserve energy production pathways. It is not known if the SRC/FAK and MEK/ERK pathways are also modulated in HRMECs, and further studies with relevant cellular models are needed. More evidence from molecular docking studies indicated that crocetin had a more stable molecular interaction with VEGFR2 than crocin (Figure 7). Crocetin docked well with the binding site of VEGFR2 by forming an essential hydrogen bone with the residue Asp1046 (Guan et al., 2015), and generating a key hydrophobic interaction with the “gate keeper” residue (Val 916) of VEGFR2 (Bold et al., 2016).

An additional important result obtained in the present study on the anti-angiogenic effects of crocetin and crocin was that crocetin was more effective than crocin, because the effective concentrations of crocetin were lower than those of crocin. Moreover, crocetin showed more stable binding patterns than crocin. To understand the different effects of crocetin and crocin, their membrane permeability, caused by carboxyl or glycosyl groups at the ends of the backbone, should be considered (Bian et al., 2020). Crocetin showed better permeative ability by penetrating intestinal mucosa, whereas crocin could not penetrate an intestinal model even at a concentration of 1000 mΜ (Lautenschlager et al., 2015). Accordingly, a pharmacokinetic study showed that crocin was hydrolyzed to crocetin through the gastro-intestinal tract, and then absorbed and detected in plasma (Xi et al., 2007). In addition, the structure-activity relationship of carotenoids (including crocetin and crocin) suggested that diverse terminal structures, such as electron-rich aromatic methyl substituent and the polyene chain structures, were responsible for the antioxidant activity of carotenoids (Kim et al., 2019). These findings, together with the fact that crocetin is more effective than crocin, suggesting that crocetin may act as an active compound with a stronger anti-angiogenic effect. However, further studies such as pharmacokinetic ones and studies of the structure-activity relationship, are needed to support this hypothesis.

Collectively, our findings provided strong evidence supporting the anti-angiogenic activities of crocetin and crocin, and indicated that crocetin had more anti-angiogenic and anti-VEGF therapeutic value for AMD.

## Data Availability

The original contributions presented in the study are included in the article/Supplementary Material, further inquiries can be directed to the corresponding author.

## Abbreviations

AMD, age-related macular degeneration; CNV, choroidal neovascularization; hpf, hours post fertilization; HPLC, high-performance liquid chromatography; HRMECs, human retinal microvascular endothelial cells; HUVECs, human umbilical vein endothelial cells; MTA, microtubule-targeting agents; RPE, retinal pigment epithelia; SIVs, subintestinal vein vessels; TBHP, tert-butyl hydrotert-butyl hydroperoxide; VEGF, vascular endothelial growth factor; VEGFR, VEGF receptor; VRI, VEGFR tyrosine kinase inhibitor II.
