# Supplementary material for: Crocetin and Its Glycoside Crocin, Two Bioactive Constituents From Crocus sativus L. (Saffron), Differentially Inhibit Angiogenesis by Inhibiting Endothelial Cytoskeleton Organization and Cell Migration Through VEGFR2/SRC/FAK and VEGFR2/MEK/ERK Signaling Pathways
Source: Front Pharmacol. 2021 Apr 30;12:675359. doi: 10.3389/fphar.2021.675359 (PMC8120304; doi:10.3389/fphar.2021.675359)
Supplement: Supplementary file 1 [file DataSheet1.docx]

Supplementary Material


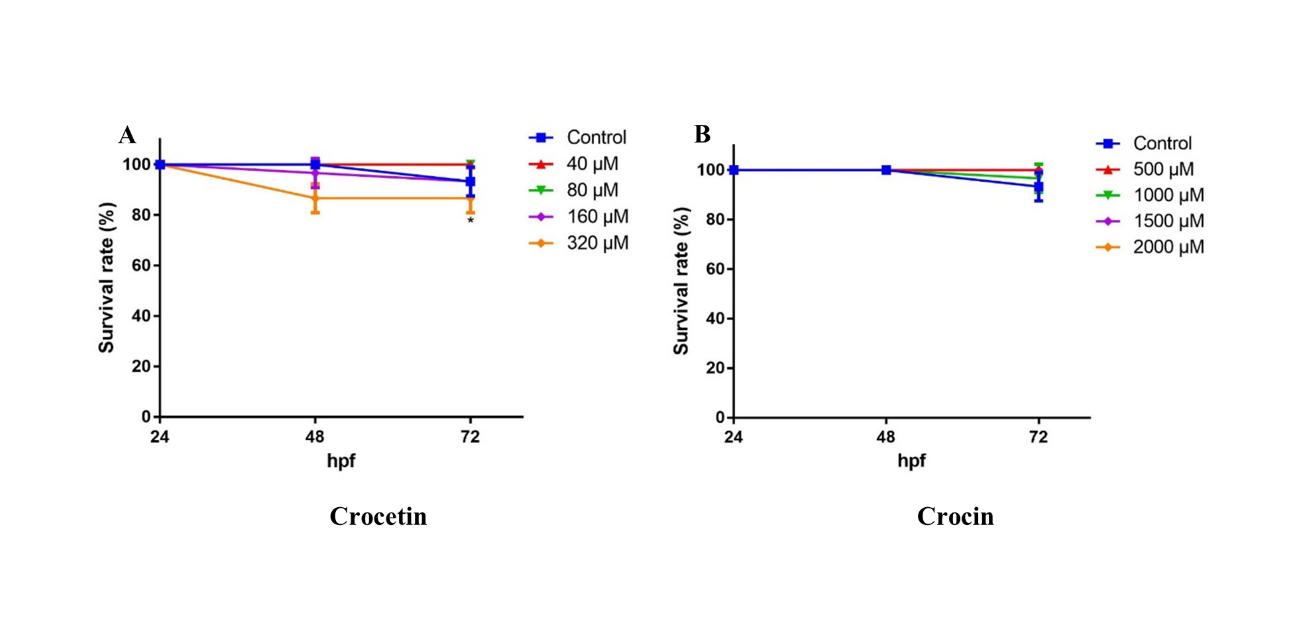


**Supplementary Figure 1.** Survival rate of zebrafish embryos after treating with different concentrations of (A) crocetin and (B) crocin. Zebrafish embryos receiving 0.1% DMSO served as a vehicle control. Data were percentage of control measured as means ± SD (10 zebrafish embryos per well from three time-independent experiments, n=3). Statistical analysis was performed by one-way ANOVA followed by the Dunnett's test. *p<0.05 versus control group.
